# Total gastrectomy increases the incidence of grade III and IV toxicities in patients with gastric cancer receiving adjuvant TS-1 treatment

**DOI:** 10.1186/1477-7819-11-287

**Published:** 2013-11-01

**Authors:** Wen-Chi Chou, Chia-Lun Chang, Keng-Hao Liu, Jun-Te Hsu, Hung-Chih Hsu, Wen-Chi Shen, Yu-Shin Hung, Jen-Shi Chen

**Affiliations:** 1 Division of Hematology-Oncology, Department of Internal Medicine, No. 5 Fuxing Street, Guishan Township, Taoyuan County 333, Taiwan ROC; 2 Department of Surgery, Linkou Chang Gung Memorial Hospital and Chang Gung University College of Medicine, Taoyuan, Taiwan

**Keywords:** Adjuvant chemotherapy, Compliance, Gastric cancer, Safety profile, TS-1

## Abstract

**Background:**

We aimed to evaluate the safety and efficacy of TS-1 adjuvant chemotherapy in Taiwanese patients with gastric cancer.

**Methods:**

We included in this study patients with locally advanced gastric cancer who received adjuvant TS-1 or 5-fluorouracil chemotherapy after curative surgery and extended lymph node dissection between 1 June 2008 and 31 December 2012 at Chang Gung Memorial Hospital. Patient characteristics, tumor features, safety profiles and compliance with TS-1 treatment were retrospectively analyzed from medical charts.

**Results:**

Forty patients received adjuvant chemotherapy with TS-1 and 193 with 5-fluorouracil within the study period. The 1- and 2-year overall survival rates were 90.6% and 87% in the TS-1 group and 95.4% and 86.8% in the 5-fluorouracil group (*P* = 0.34). The 1- and 2-year disease-free survival rates were 90.6% and 74.7% in the TS-1 group and 88% and 75.7% in the 5-fluorouracil group (*P* = 0.66). In the TS-1 group, tumor recurrence was more frequent in those with >15 metastatic lymph nodes than ≤15. Overall, 78.9%, 74.3%, 62.1% and 56% of patients underwent TS-1 treatment for at least 3, 6, 9 and 12 months, respectively. The most common adverse events of TS-1 were skin hyperpigmentation (55%), diarrhea (27.5%), dizziness (27.5%) and leucopenia (20%). Severe adverse events (SAEs; grade III or IV toxicity) were diarrhea (7.5%), stomatitis (7.5%), leukopenia (5%), vomiting (2.5%), anorexia (2.5%) and dizziness (2.5%). Patients who underwent total gastrectomy had a significantly greater risk of TS-1-related SAEs than patients who underwent subtotal gastrectomy (40% versus 8%, *P* = 0.014).

**Conclusions:**

The incidence of SAEs during TS-1 therapy was more common in Taiwanese patients with gastric cancer who underwent total gastrectomy compared with those who underwent subtotal gastrectomy. Clinicians must be aware of and able to manage these SAEs to maximize patient compliance with adjuvant TS-1.

## Background

Gastric cancer is the fourth most common cancer and the second most common cause of cancer death worldwide. In 2008, there were an estimated 989,600 new gastric cancer cases and 738,000 deaths worldwide [1]. In Taiwan, gastric cancer is the sixth leading cause of cancer-related mortality, causing approximately 2,446 patient deaths in 2009 [2]. Surgical resection is the only curative modality for gastric cancer. Extended (D2) lymph node dissection is considered a standard modality for curative gastrectomy among experienced surgeons worldwide [3-5]; however, approximately 25% to 40% of patients experience tumor recurrence within 5 years after surgery [3-5]. To overcome this dilemma, the role of adjuvant chemotherapy in the treatment of patients with resectable gastric cancer should be addressed.

Gastric cancer is relatively sensitive to chemotherapy in its advanced stages [6,7]; thus, adjuvant chemotherapy is often administered to patients with gastric cancer after curative surgery to prevent tumor recurrence and prolong survival time. Unfortunately, early clinical trials with various adjuvant chemotherapy regimens produced negative results [8-15]. However, data from meta-analysis studies [16,17] indicated a significant survival benefit associated with fluorouracil-based adjuvant chemotherapy. There was no consensus regarding the optimal adjuvant chemotherapeutic regimen, schedule or duration of treatment for gastric cancer until the results of three recently published phase III studies became available after 2007 [18-20]. Tegafur-uracil was the first agent demonstrated to prolong both overall survival and relapse-free survival compared to surgery alone in a large phase III randomized study [18]. This study was terminated before the target number of patients was reached because accrual was slower than expected and the benefit was limited in patients with serosa-negative locally advanced gastric cancer classified according to the Japanese staging system [21].

The Adjuvant Chemotherapy Trial of TS-1 for Gastric Cancer (ACTS-GC) was the first well-designed, large-enrollment phase III study that showed significant benefit of adjuvant chemotherapy for Japanese patients with stage II and III gastric cancer who had undergone D2 dissection [19]. Among patients who underwent 1 year of treatment with TS-1, an oral fluoropyrimidine, the 3-year overall survival rate was 80%, compared to 70.1% in the surgery alone group (*P* <0.001). The survival benefit persisted at 5 years [22]. The latest phase III study examining capecitabine and oxaliplatin combination treatment (CLASSIC study) in stage II to III stomach cancer after D2 resection [20] was reported in January 2012. The primary end-point (3-year disease-free survival) was achieved in 74% of patients in the chemotherapy group and in 59% of patients in the surgery alone group (*P* <0.0001). However, 3-year overall survival was not significantly different between the two groups (83% versus 78%; *P* = 0.0493). Thus, a longer follow-up period may be needed to demonstrate the potential survival benefit of capecitabine and oxaliplatin combination treatment.

Until now, TS-1 has been the only effective adjuvant chemotherapy agent known to prolong disease-free survival and overall survival in patients with stage II and III gastric cancer. TS-1 has been available in Taiwan since 2007. Depending on the patient’s preference and the clinician’s decision, TS-1 could be used as an adjuvant treatment for patients with gastric cancer after curative D2 dissection in clinical practice. This study aimed to evaluate the safety and efficacy of TS-1 as adjuvant chemotherapy for the treatment of gastric cancer in Taiwanese populations.

## Methods

### Patient selection

We retrospectively selected patients with gastric cancer who received TS-1 as adjuvant chemotherapy after curative gastrectomy between 1 June 2008 and 31 December 2012 at Chang Gung Memorial Hospital (Linkou branch), Taoyuan, Taiwan. The decision on performing either a total or subtotal gastrectomy was determined by the surgeon, based on tumor location, histological type and free resection margin. All patients achieved microscopic tumor clearance (R0), underwent resection with D2 lymph node dissection, and received TS-1 in an adjuvant setting. Patients with a histological diagnosis other than adenocarcinoma or poorly differentiated carcinoma and those who received other antitumor treatments (radiotherapy or other chemotherapeutic agents) were excluded from the analysis. Information regarding demographic data, tumor stage, tumor histology, surgery method and adverse events during TS-1 treatment was retrospectively obtained from medical charts. Patients diagnosed with gastric cancers who underwent curative gastrectomy and D2 lymph node dissection, and received intravenous 5-fluorouracil (5FU) adjuvant therapies in the same time period were retrospectively included to compare the efficacy between different adjuvant chemotherapies. The tumor stage was determined according to the American Joint Committee on Cancer (AJCC) Cancer Staging Manual (6th edition) [23]. The grading of adverse events was performed according to the Common Terminology Criteria for Adverse Events, version 3.0. All patients were followed-up until 31 December 2012. The study was approved by the ethics committee of the institute.

### Adjuvant chemotherapy treatment

When possible, patients would receive adjuvant treatment within 6 weeks after surgery. For TS-1 administration, patients with a body surface area <1.25 m^2^ received 80 mg daily, those with a body surface area ≥1.25 m^2^ and <1.5 m^2^ received 100 mg daily, and those with a body surface area of ≥1.5 m^2^ received 120 mg daily. A TS-1 capsule was given orally twice daily from day 1 to day 28 for 42 days per cycle. The planned treatment duration was nine cycles. Dose escalation or schedule modification occurred depending on the clinician’s decision to minimize adverse effects. The TS-1 compliance at each given time was determined by the following formula: (number of patients who received TS-1 treatment - number of patients who withdrew at the given time) ∕ number of patients who received TS-1 treatment at the given time period. Patients who withdrew from TS-1 treatment owing to tumor relapse were excluded in the TS-1 compliance calculation for any given time.

In Taiwan, an intravenous 5FU-based regimen is the standard adjuvant chemotherapy for patients with gastric cancer because of its efficacy, and it is covered by the National Health Insurance [24]. In our institute, adjuvant treatment for gastric patients followed the Roswell Park regimen [25], which is detailed as six courses of intravenous infusion of 5FU 500 mg/m^2^ and leucovorin 500 mg/m^2^ over a 2-hour period every week within 8 weeks per cycle. The planned completion of treatment was a total of four cycles.

### Statistical analysis

SPSS 17.0 (SPSS Inc., Chicago, IL, USA) was used for statistical analysis. Clinical characteristics data were summarized as n (%) for categorical variables and medians with ranges for continuous variables. Patients’ demographic data and the frequency of grade III and IV toxicity was tabulated as n (%) by clinical variables, and the data were compared using the chi-squared (*χ*^2^) test, or the Fisher’s exact test if the number in any cell was less than five. Overall and disease-free survival times were calculated from the date of surgery to the date of the event. Survival rates were calculated using the Kaplan-Meier method. Univariate analysis of survival for all clinical characteristics was performed using the log-rank test and Cox’s proportional hazard model. Data of patients who were lost to follow-up were censored for the analysis of overall and disease-free survival. All statistical assessments were considered significant at *P* <0.05.

## Results

Our study included 40 patients who received adjuvant chemotherapy with TS-1 and 193 with 5FU. Their basic demographic data are summarized in Table 1. In the TS-1 group, the median age was 61 years (range, 27 to 88), and 60% (24) were men. Thirty-nine patients (96%) had an excellent Eastern Cooperative Oncology Group performance status (grade 0 or 1). Diffuse-type and intestinal-type gastric cancers were noted in 25 and 15 patients, respectively. Fifteen patients underwent total gastrectomy, and the remaining 25 patients underwent subtotal gastrectomy. There was no significant difference in patients received TS-1 treatment between types of curative surgery they received when comparing each subcategory of age, gender, tumor histological type, and T or N stage of the sixth edition AJCC staging system (data not shown). Based on the AJCC staging system (sixth edition), five patients were at stage Ib, seventeen were at stage II, eight were at stage IIIa, two were at stage IIIb, and eight were at stage IV. All stage IV patients were M0 status, five patients had N3 diseases, two had T4 diseases and one patient had N3 and T4 disease. There was no significant difference in clinical characteristics between the TS-1 and 5FU treatment groups.

**Table 1:** Clinical characteristics of patients with gastric cancer receiving adjuvant TS-1 and 5-fluorouracil chemotherapy

	**TS-1 adjuvant group, n (%)**	**5-fluorouracil adjuvant group, n (%)**	***p *** **value**
**Total**	40 (100)	193 (100)	
**Male gender**	24 (60)	123 (63.7)	0.39
**Median age (range)**	61.0 (27 to 88)	61.1 (27 to 90)	0.63
**ECOG performance status scale**			
0	32 (80)	133 (68.9)	0.24
1	7 (17.5)	47 (24.4)	
2	1 (2.5)	13 (6.7)	
**Histological type**			
Intestinal	15 (37.5)	61 (31.6)	0.29
Diffuse	25 (62.5)	132 (68.4)	
**Gastrectomy method**			
Subtotal	25 (62.5)	124 (64.2)	0.48
Total	15 (37.5)	69 (35.8)	
**TNM stage (AJCC, 6**^**th**^**)**			
Ib	5 (12.5)	48 (24.9)	0.12
II	17 (42.5)	46 (23.8)	
IIIa	8 (20)	51 (26.4)	
IIIb	2 (5)	13 (6.7)	
IV	8 (20)	35 (18.1)	
**Tumor stage (AJCC, 6**^**th**^**)**			0.14
T1	6 (15)	45 (23.3)	
T2	22 (55)	68 (35.2)	
T3	9 (22.5)	59 (30.6)	
T4	3 (7.5)	21 (10.9)	
**Nodal stage (AJCC, 6**^**th**^**)**			
N0	2 (5)	28 (14.5)	0.28
N1	23 (57.5)	97 (50.3)	
N2	9 (22.5)	50 (25.9)	
N3	6 (15)	18 (9.3)	

AJCC, American Joint Committee on Cancer; ECOG, Eastern Cooperative Oncology Group; TNM, Tumor-node-metastasis.

By the end of December 2012, the median follow-up duration was 627 days (range, 178 to 1,711) in TS-1 treatment group. Eight patients experienced tumor recurrence, six of whom during treatment and two after treatment completion. Four of these patients died from cancer-related mortality (morbidities) at the end of the study. No treatment-related mortality was observed.

**Figure 1:**
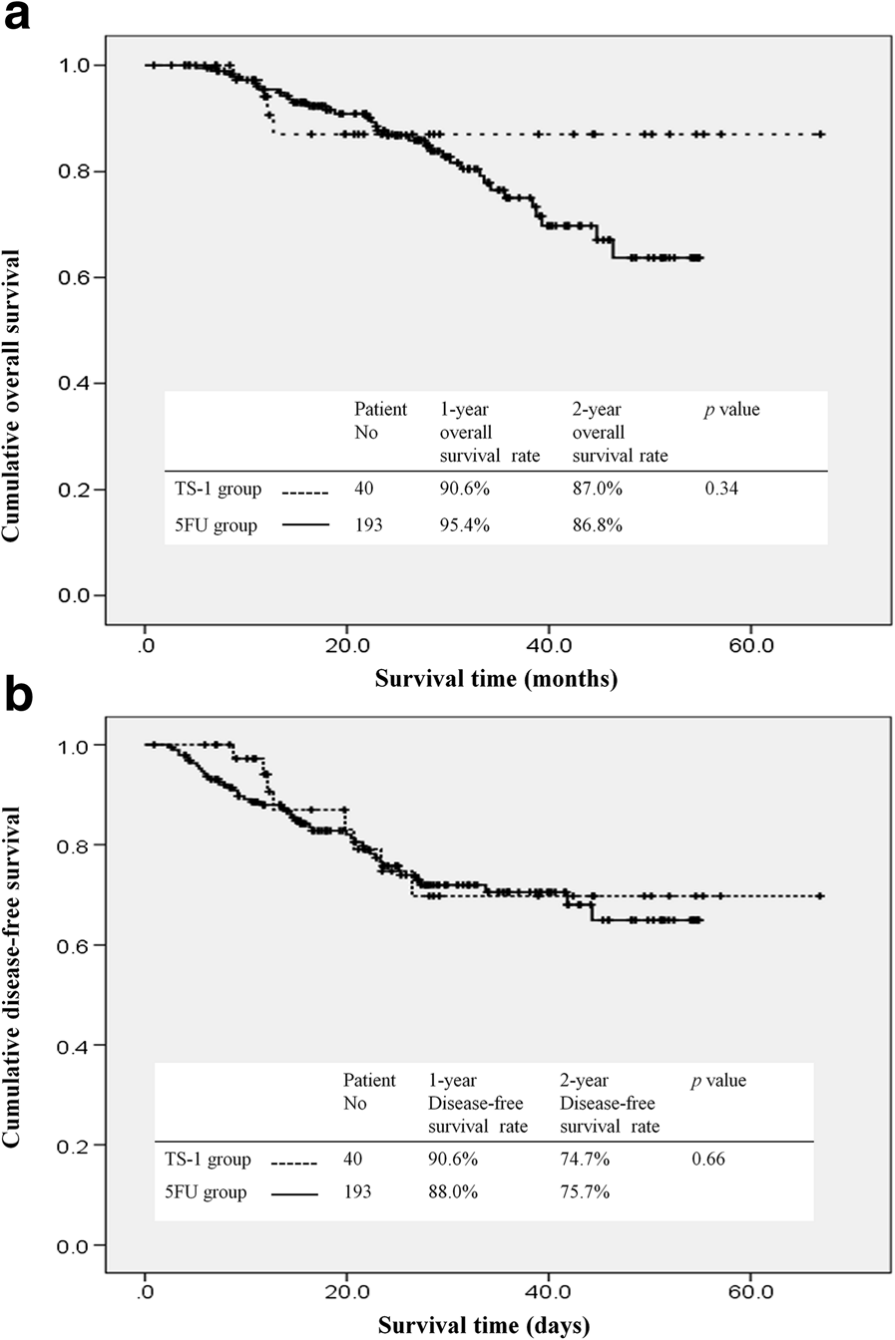
**Survival rates of patients with gastric cancer receiving either TS-1 or 5-fluorouracil adjuvant treatment. (a)** Cumulative overall survival and **(b)** disease-free survival.

The cumulative overall and disease-free survival curves of patients receiving adjuvant treatment with either TS-1 or 5FU are presented in Figure 1. The 1- and 2-year overall survival rates were 90.6% and 87% in the TS-1 group and 95.4% and 86.8% in the 5FU group. There was no significant difference in overall survival (*P* = 0.34) and disease-free survival (*P* = 0.66) between the TS-1 and 5FU group.

Patient compliance with TS-1 is summarized in Table 2. The median TS-1 treatment duration was 226 days (range, 7 to 400). TS-1 schedule modification was noted in 18 patients, and six patients underwent TS-dose de-escalation. Nine patients continued with TS-1 treatment until the end of the study. The reasons for discontinuation included completion of TS-1 treatment course (14 out of 31, 45%), toxicity (9 out of 31, 29%), recurrent cancer (6 out of 31, 19%) and economic factors (2 out of 31, 6%). Overall, 78.9%, 74.3%, 62.1% and 56% of patients continued TS-1 treatment for at least 3, 6, 9 and 12 months, respectively.

**Table 2:** TS-1 dose modification and compliance of patients with gastric cancer

	**n (%)**
Received treatment schedule modification	18 (45)
Underwent dose de-escalation	6 (15)
Discontinued TS-1 treatment at study end	31 (77.5)
**Reasons for discontinuation**	
Completion of TS-1 treatment	14 out of 31 (45)
Toxicity	9 out of 31 (29)
Tumor recurrence	6 out of 31 (19)
Economic factors	2 out of 31 (6)
**TS-1 treatment duration (compliance)**	
At least 3 months	30 out of 38 (78.9)
At least 6 months	26 out of 35 (74.3)
At least 9 months	18 out of 29 (62.1)
Completed nine full cycles	14 out of 25 (56)

The median TS-1 treatment duration was 226 days (range 7 to 400). TS-1 compliance: the number of patients who discontinued treatment because of toxicity or economic factor subtracted from the number of patients who underwent TS-1 treatment at the given time. Patients who withdrew from TS-1 treatment owing to tumor relapse were excluded in the TS-1 compliance calculation for any given time.

We assessed the incidence of adverse events associated with TS-1 in all patients (n = 40; Table 3). The most common adverse events were skin hyperpigmentation (55%), diarrhea (27.5%), dizziness (27.5%) and leucopenia (20%). Grade III adverse events included diarrhea (5%), stomatitis (7.5%), leukopenia (5%), vomiting (2.5%), anorexia (2.5%) and dizziness (2.5%). Only one grade IV adverse event was observed in this study (diarrhea, 2.5%).

**Table 3:** Adverse events experienced by patients receiving adjuvant TS-1 treatment

**Events**	**Patients, n = 40 (%)**				
	**Grade I**	**Grade II**	**Grade III**	**Grade IV**	**Total**
Skin pigmentation	18 (45)	4 (10)	0	0	22 (55)
Diarrhea	1 (2.5)	7 (17.5)	2 (5)	1 (2.5)	11 (27.5)
Fatigue	1 (2.5)	2 (5)	0	0	3 (7.5)
Vomiting	3 (7.5)	2 (5)	1 (2.5)	0	6 (15)
Stomatitis	0	2 (5)	3 (7.5)	0	5 (12.5)
Anorexia	5 (12.5)	1 (2.5)	1 (2.5)	0	7 (17.5)
Alopecia	3 (7.5)	0	0	0	3 (7.5)
Dizziness	8 (20)	2 (5)	1 (2.5)	0	11 (27.5)
Leukopenia	1 (2.5)	5 (12.5)	2 (5)	0	8 (20)
Thrombocytopenia	2 (5)	0	0	0	2 (5)
Anemia	1 (2.5)	4 (10)	0	0	5 (12.5)

Table 4 summarizes predictive factors for 2-year disease-free survival and grade III or IV toxicity in patients receiving TS-1. There was a significant difference between patients with >15 metastatic lymph nodes (N3 stage) and ≤15 metastatic lymph nodes (N0 to N2 stage) in their 2-year disease-free survival (17% versus 91%; hazard ratio 0.09; 95% confidence interval 0.02, 0.40; *P* <0.001).

**Table 4:** Predictive factors for 2-year disease-free survival rate and grade III or IV toxicity in patients with gastric cancer who underwent adjuvant TS-1 treatment

**Variables**		**2-year disease-free survival rate**	***P***	**Grade III or IV toxicity n (%)**	***P***
Gender	Male (n = 24)	75%	0.68	6 (25.0)	0.33
	Female (n = 16)	74%		2 (12.5)	
Age	≥65 (n = 13)	82%	0.29	1 (7.7)	0.18
	<65 (n = 27)	50%		7 (25.9)	
Histological type	Diffuse type (n = 25)	74%	0.57	6 (24.0)	0.41
	Intestinal type (n = 15)	75%		2 (13.3)	
ECOG performance status scale	0 (n = 32)	74%	0.63	6 (18.8)	0.74
	1 to 2 (n = 8)	75%		2 (25.0)	
Type of gastrectomy	Total (n = 15)	72%	0.66	6 (40.0)	0.014
	Subtotal (n = 25)	76%		2 (8.0)	
T stage	1 to 2 (n = 28)	82%	0.44	6 (21.4)	0.73
	3 to 4 (n = 12)	75%		2 (16.7)	
Lymph node metastases numbers	0 to 15 (n = 34)	91%	<0.001	0 (0)	0.18
	>15 (n = 6)	17%	(HR 0.09, 95% CI 0.02, 0.40)	8 (23.5)	

CI: confidence interval; ECOG, Eastern Cooperative Oncology Group; HR, hazard ratio.

Patients who underwent total gastrectomy had a significantly greater risk of grade III or IV toxicities associated with TS-1 compared with patients who underwent subtotal gastrectomy (40% versus 8%, *P* = 0.014). There was no difference in 2-year disease-free survival and serious adverse events in the TS-1 treatment group when comparing between patients’ age, gender, performance status scale, cancer histological type or tumor stage.

## Discussion

In this study, we collected data from a single institution regarding compliance, adverse events and efficacy of adjuvant TS-1 therapy for Taiwanese populations with gastric cancer after curative surgery and D2 lymph node dissection. Although the number of patients included was small, the data are representative of patients with gastric cancer treated with adjuvant TS-1 therapy. TS-1 adjuvant treatment efficacy is comparable to a Roswell Park regimen in terms of overall survival and disease-free survival.

The majority of adverse events associated with TS-1 were mild and manageable in this study; toxicities of the gastrointestinal tract (diarrhea (7.5%), stomatitis (7.5%) and vomiting (2.5%)) and hematologic toxicity with leukopenia (5%) constituted the most common grade III and IV toxicities related to TS-1. In the ACTS-GC study, the most common grade III and IV toxicities were anorexia (6%), nausea (3.7%) and diarrhea (3.1%); only 1.2% of patients experienced grade III leukopenia. A diverse array of toxicities related to TS-1 has been reported among different ethnicities. Two phase I studies of TS-1 concluded that myelosuppression was the most severe dose-related toxicity and recommended an 80 to 120 mg daily dose of TS-1 in Japanese patients [26,27]. Four phase I studies from the US and the Netherlands concluded that diarrhea was the most severe dose-related toxicity and recommended a TS-1 dose of 50 to 80 mg in Caucasian populations [28-31]. A larger 5FU area under the curve was noted in Caucasian patients compared with Japanese patients given the same TS-1 dose, which partially explained the different toxicity profiles [32]. The pharmacokinetic profiles of TS-1 in Taiwanese populations were comparable with those from Japanese and other Asian populations [26,27]. The minor difference in toxicity profiles between the current study and the ATCS-GC study may be explained by historical comparison and retrospective bias in data collection in our study. Our findings indicate that in Asian patients who receive daily TS-1 treatment, clinicians should pay attention to gastrointestinal toxicity, including diarrhea, stomatitis and vomiting, and should manage them early and properly with medications and dose or schedule modification.

Patient compliance with TS-1 throughout the duration of treatment was 4% to 8% lower than what was reported throughout the duration of treatment in the ACTS-GS study [26]. The difference was acceptable and expected when comparing data from clinical practice with clinical study [33]. A subgroup analysis [34] from the ACTS-GC study demonstrated that overall and disease-free survival times were better in patients who completed 12 months of TS-1 adjuvant treatment. However, the factors related to lack of compliance with adjuvant TS-1 chemotherapy were not well explored. Recent studies by Aoyama *et al*. reported that weight loss after surgery (a decrease of less than 15%) and creatinine clearance (lower than 60 mL/min) were factors related to discontinuation of TS-1 treatment [35,36]. Tsujimoto *et al*. reported that younger patient age and treatment by a senior doctor (with more than 15 years of experience) were factors associated with successful completion of 12 months of TS-1 adjuvant treatment [34].

In the current study, the main reason for early withdrawal from TS-1 treatment was adverse events. Patients who underwent total gastrectomy experienced a significantly higher incidence of grade III or IV toxicities under TS-1 therapy in this study. A Korean study that included 305 patients with gastric cancer undergoing TS-1 treatment in the same setting showed that total gastrectomy was associated with a higher risk of hematologic grade III and IV toxicities than distal gastrectomy; however, there was no difference in non-hematologic toxicity [37]. Because of the rarity of hematologic toxicity and the limited number of patients, we were unable to address the relationship between hematologic toxicity and total gastrectomy in this study. There were no differences in the pharmacokinetic profile of TS-1 components in 12 Taiwanese patients with advanced gastric cancer, regardless of whether they underwent gastrectomy [38]. Kinoshita *et al*. observed a slightly higher incidence of adverse reactions in adjuvant TS-1 treatment compared with that in unresectable or recurrent gastric cancer - the authors concluded the difference was probably due to the influence of gastrectomy [39]. However, data regarding the pharmacokinetic profiles of TS-1 components in patients who underwent total gastrectomy or subtotal gastrectomy were lacking. The reason for the higher incidence of serious adverse events in patients who underwent total gastrectomy and received TS-1 treatment was unknown. Because of inconsistency in clinical experience, it is unclear whether total gastrectomy alters the pharmacokinetic profile of TS-1. An understanding of the impact of total gastrectomy on TS-1 treatment will require further study.

In our study, lymph node stage had a significant impact on 2-year disease-free survival, especially in patients with more than 15 metastatic lymph nodes. In the ACTS-GC study, less than 6% of enrolled patients had more than 16 metastatic lymph nodes. The impact of lymph node metastases on overall survival in TS-1-treated patients was limited to those with N0 and N1 nodal stage in subgroup analysis [22]. In the CLASSIC study, subgroup analysis showed that the greatest chemotherapy benefit was in patients with N1 and N2 nodal stage [20]. Considering the currently available evidence, adjuvant TS-1 should be considered as the treatment of choice for patients with gastric cancer with fewer than eight metastatic lymph nodes, whereas capecitabine and oxaliplatin should be the treatment of choice for patients with 1 to 15 metastatic lymph nodes. In line with the ACTS-GC study, our study does not lead us to recommend adjuvant TS-1 treatment for patients with gastric cancer who have >15 metastatic lymph nodes.

The current study had some limitations. First, we were only able to include a small number of patients. With a limited follow-up period and a non-randomized design in comparison to adjuvant 5FU treatment, the efficacy of TS-1 in treating gastric cancer in the adjuvant setting could not be accurately addressed. Being a novel agent, treatment with TS-1 is not yet covered by the National Health Insurance in Taiwan. Thus, patients are responsible for paying the fee. The price for a 20 mg pill of TS-1 is US$11.40. The cost becomes US$11,525 to US$17,287 assuming a dose of 80 to 120 mg daily over the full duration of treatment. Because of this financial burden, few patients are able to receive TS-1 as adjuvant chemotherapy for gastric cancer treatment. Second, based on a retrospective data collection in the current study, the safety profile of TS-1 was under-assessed, and because only a small number of patients was included, the incidence of severe adverse effects due to TS-1 could not be evaluated. In the future, nationwide studies are necessary to address these limitations. As more results become available from various clinical trials focusing on different solid tumors, clinicians and patients will become more informed and familiar with the safety profile of TS-1.

## Conclusions

TS-1 is easy to administer on an outpatient basis and is the only adjuvant regimen shown to improve overall survival and disease-free survival in patients with gastric cancer. Diarrhea, stomatitis and vomiting were the most common grade III and IV toxicities in patients with gastric cancer who were receiving adjuvant TS-1. The incidence of grade III and IV toxicities associated with TS-1 was more common in patients who underwent total gastrectomy. Clinicians must have the awareness and knowledge to manage these grade III and IV toxicities to maximize patient compliance with adjuvant TS-1.

## Abbreviations

5FU: 5-fluorouracil; ACTS-GC: Adjuvant Chemotherapy Trial of TS-1 for Gastric Cancer; AJCC: American Joint Committee on Cancer; D2: Extended (lymph node dissection).

## Competing interests

The authors declare that they have no competing interests.

## Authors’ contributions

CWC, CLC, LKH, HJT and HHC participated in the design of the study and draft the manuscript. SWC and HYS and participated data collection and performed the statistical analysis. CJS conceived of the study. All authors read and approved the final manuscript.
