# Correction: Total gastrectomy increases the incidence of grade III and IV toxicities in patients with gastric cancer receiving adjuvant TS-1 treatment

**DOI:** 10.1186/1477-7819-11-310

**Published:** 2013-12-17

**Authors:** Wen-Chi Chou, Chia-Lun Chang, Keng-Hao Liu, Jun-Te Hsu, Hung-Chih Hsu, Wen-Chi Shen, Yu-Shin Hung, Wei Hong Cheng, Jen-Shi Chen

**Affiliations:** 1 Division of Hematology-Oncology, Department of Internal Medicine, Linkou Chang Gung Memorial Hospital and Chang Gung University College of Medicine, Taoyuan, Taiwan; 2 Department of Surgery, Linkou Chang Gung Memorial Hospital and Chang Gung University College of Medicine, Taoyuan, Taiwan

## Correction

After publication of this work [1], we noted that we inadvertently failed to include the complete list of all coauthors. Wei Hong Cheng has now been added to the list of authors and the Authors’ contributions and Competing interests section modified accordingly.

## Competing interests

The authors declare that they have no competing interests.

## Authors’ contributions

CWC, CLC, LKH, HJT and HHC participated in the design of the study and draft the manuscript. SWC, HYS and CWH participated data collection and performed the statistical analysis. CJS conceived of the study. All authors read and approved the final manuscript.
